# Dairy Intakes at Age 10 Years Do Not Adversely Affect Risk of Excess Adiposity at 13 Years^1^,^2^,^3^

**DOI:** 10.3945/jn.113.183640

**Published:** 2014-04-17

**Authors:** Sherman J. Bigornia, Michael P. LaValley, Lynn L. Moore, Kate Northstone, Pauline Emmett, Andy R. Ness, P. K. Newby

**Affiliations:** 4 Department of Pediatrics,; 5 Section of Preventive Medicine and Epidemiology, Department of Medicine, and; 6 Division of Graduate Medical Sciences, Boston University School of Medicine, Boston, MA; Departments of; 7 Biostatistics and; 8 Epidemiology, Boston University School of Public Health, Boston, MA;; 9 School of Social and Community Medicine and; 10 School of Oral and Dental Sciences, University of Bristol, Bristol, UK; and; 11 Program in Gastronomy, Culinary Arts, and Wine Studies, Boston University Metropolitan College, Boston, MA

## Abstract

Evidence of an association between milk intake and childhood adiposity remains inconsistent, with few data available regarding the effects of the amount of dairy fat consumed. This study examined the relation between dairy consumption (total, full, and reduced fat) at age 10 y on risk of excess adiposity at age 13 y in participants of the Avon Longitudinal Study of Parents and Children (ALSPAC; *n* = 2455). Intakes were assessed by 3-d dietary records. Total body fat mass (TBFM) using dual-energy X-ray absorptiometry was examined at 13 y. Outcomes included excess TBFM (top quintile of TBFM), overweight, and change in body mass index (BMI). The highest vs. lowest quartile of total dairy consumers (g/d) at age 10 y did not have an increased risk of excess TBFM (OR: 0.73; 95% CI: 0.46, 1.16; *P*-trend = 0.28) or overweight (OR: 0.69; 95% CI: 0.41, 1.15; *P* = 0.24) at age 13 y. Children in the highest quartile of full-fat dairy intakes vs. those in the lowest quartile had a reduced risk of excess TBFM (OR: 0.64; 95% CI: 0.41, 1.00; *P* = 0.04) and a suggestion of a reduction in overweight (OR: 0.65; 95% CI: 0.40, 1.06; *P* = 0.19) at age 13 y. Furthermore, the highest vs. lowest consumers of full-fat products had smaller gains in BMI during follow-up [2.5 kg/m^2^ (95% CI: 2.2, 2.7) vs. 2.8 kg/m^2^ (95% CI: 2.5, 3.0); *P* < 0.01]. Associations with reduced-fat dairy consumption did not attain statistical significance. In this study, dairy consumption was not related to excess fat accumulation during late childhood. Estimates had wide confidence limits but generally showed inverse relations between dairy intakes and risk of excess adiposity. Additional prospective research is warranted to confirm the effects of dairy intake on obesity in children.

## Introduction

Worldwide, 43 million children aged 0 to 5 y are overweight or obese (1), and in the United States, current estimates among 2- to 19-y-olds range from 23% to 33% (2). The burden of overweight and obesity is of great concern because childhood adiposity affects cardiometabolic risk, including high blood pressure (3), as well as adulthood morbidity and mortality (3–5). A core component of any strategy to manage and prevent childhood obesity is dietary modification. Therefore, it is important to identify dietary targets that have the potential to help limit excess weight gain in children.

Over the same time period that the prevalence of obesity has increased, children’s dairy consumption, particularly milk (6, 7), has decreased along with fruit and vegetable intakes (8) and physical activity amounts (9). With the use of nationally representative U.S. survey data, Popkin (6) found that, from 1997 to 2006, per capita daily energy intake of whole milk among children decreased by 46%. Interestingly, low-fat milk intake increased over the same time period, although it remained a small fraction of total milk consumption (6). Similarly, from 1986/1987 to 2008/2009, reductions in whole-milk intakes among children and adolescents in Great Britain were reported (7). Dairy is composed of a variety of bioactive compounds that may protect against the accumulation of excess fat mass (10–13); conversely, it contributes energy and contains hormones that may promote weight gain (14). The current literature on the relation between dairy consumption and adiposity in children and/or adolescents is inconsistent (15–17). In a 2005 review of observational studies and randomized controlled trials, Huang and McCrory (15) concluded that little evidence supports a protective effect of dairy intake on risk of childhood obesity; these results were reiterated in more recent reviews (16, 17).

Few previous longitudinal studies have examined the relations between full- and reduced-fat dairy foods and excess weight gain during childhood (16). In light of the discrepant findings regarding the association between total dairy intake and adiposity among children and the paucity of evidence on the effects by amount of dairy fat, more research is warranted to understand these relations. Therefore, our aims were to determine the effects of total and full- and reduced-fat dairy intake in children at 10 y of age on risk of excess total body fat mass (TBFM) and overweight at age 13 y. We hypothesized that total and full- and reduced-fat dairy consumption in preadolescence would not be associated with excess fat accumulation in early adolescence.

## Participants and Methods

The Avon Longitudinal Study of Parents and Children (ALSPAC) is a population-based, prospective cohort study designed to examine the relation between the environment and the health and development of children (18). All pregnant women residing in the former County of Avon located in southwest England with expected delivery dates between April 1991 and December 1992 were eligible (*n* = 20,248) (18). From this sample, 14,541 women were enrolled. At 7 y of age, children in the study were invited to attend clinical examinations at which time more detailed information was obtained. At various ages, data were collected on diet, anthropometry, body composition by DXA, and physical activity. Anthropometric measurements were obtained at ages 10, 11, and 13 y and body fat mass by DXA at 11 and 13 y. We excluded participants if they were missing anthropometric, DXA, dietary, and/or physical activity information (used to derive dietary reporting errors; description to follow).

In this study, anthropometric and DXA measures were available for 5102 children, and 2455 of them had dietary and physical activity information. At baseline (10 y), we found that participants missing dietary and physical activity data (*n* = 2647), compared with those with complete data, were ∼0.3 ± 2.7 mo older (*P* < 0.0001). Baseline height (*P* = 0.70) and BMI (*P* = 0.89) and the prevalence of overweight (*P* = 0.57) and maternal overweight (*P* = 0.44) were not different between groups. Children with complete data had a larger proportion of mothers with a high level of education (A level or university degree) compared with those who were missing information (49% vs. 42%; *P* < 0.0001; data not shown). The ALSPAC Law and Ethics Committee and the local research ethics committees and the Institutional Review Board of Boston University Medical Center approved this study. Parents provided informed consent at the time of enrollment and for any additional measures.

### Dietary assessment and dairy consumption.

Participants, with parental assistance as needed, were asked to complete 3-d dietary records before the 10-y and 13-y clinic visits. The completeness of food records was assessed by trained nutrition field-workers at the clinic visits. Software developed by the Medical Research Council Human Nutrition Research Unit in Cambridge, United Kingdom (19), was used to assign food codes and weights to all foods and beverages. Average daily nutrient intakes were estimated by BRIGADE, a nutrient analysis program based on the 5th edition of McCance and Widdowson’s food tables and supplements (20). Information not available in the nutrient databank was obtained from the National Diet and Nutrition Survey nutrient databases, manufacturers’ information, and/or recipe calculations.

The definition of a dairy product was consistent with that for the USDA (21) and included white milk (cow, sheep, and goat), flavored milk, cheese, yogurt, ice cream made with dairy, and other dairy desserts (e.g., pudding). Cheese included that eaten on its own and that consumed on a sandwich. Dairy was further categorized on the basis of fat content. Reduced-fat dairy products included those made with semiskimmed (1.7%) or skimmed milk and any reduced-fat cheese (including cottage cheese) or yogurt product. Dairy products made with whole milk were categorized as full fat. Because of methodologic constraints of the dietary database, milk products from mixed dishes (e.g., cheese on pizza) were not included in the calculation of dairy.

For our analyses, we first considered dairy in grams per day and then in servings per day. In line with the USDA’s definition (21) and consistent with other studies (22, 23), a serving of dairy was defined as the calcium equivalent to 1 cup of milk, which is equal to ∼1 cup of yogurt, 1.5 ounces of a hard cheese (e.g., cheddar), and 1 cup of dairy dessert (e.g., pudding) (21). We first examined dairy in grams vs. calcium-equivalent servings for several reasons. First, ALSPAC is a UK-based population cohort study, where dairy portions are recommended in grams (24) and not cups and ounces as in the United States (21). Second, calcium is not the only dairy component believed to protect against excess adiposity. Other bioactive factors include dairy proteins (10) and lipids (12, 25, 26), which contribute largely to dairy weight.

### Adiposity and outcome measures.

The same protocols were used at each visit to assess adiposity. Weight was measured to the nearest 0.2 kg by using a Tanita Body Fat Analyzer (model TBF 305) and height was measured to the nearest millimeter by using a Harpenden stadiometer (Holtain). BMI was calculated as weight (kg) divided by height squared (m^2^). TBFM (in kg) was obtained by DXA using a Lunar Prodigy narrow fan beam densitometer (GE Medical Systems Lunar). The outcomes of interest were overweight and excess fat mass at age 13 y. We defined overweight or obese by using BMI (kg/m^2^) in accordance with the International Obesity Taskforce age- and sex-specific cutoffs that match adult cutoff values of 25 kg/m^2^ (overweight) and 30 kg/m^2^ (obese) at 18 y of age (27). Because a standard definition of overweight using TBFM in children does not exist, we developed our own definition for this study in which children in the top 20% of TBFM were categorized as overweight (excess TBFM). TBFM was adjusted for age and height separately by sex, consistent with previous work conducted with the use of ALSPAC data (28). In our sample, the prevalence of overweight, using age- and sex-specific BMI cutoffs (27), was 20.7% and 19.7% at ages 10 and 13 y, respectively. The use of a top 20% cutoff to describe excess TBFM was appropriate because it was close to BMI-specific overweight prevalences and provided a relatively large sample of cases.

### Physical activity and dietary reporting errors.

Physical activity was collected at age 13. Children wore a uniaxial accelerometer (Actigraph) around their waist during waking hours for 7 d (29), which measured activity in counts per minute. Participants with ≥3 d of at least 10 h of data daily were used in all analyses. Dietary reporting errors were assessed at age 13 y when both diet and physical activity were collected at the same time point. As described elsewhere (30), objectively measured physical activity amounts were applied to physical activity coefficients in energy requirement equations as part of methods developed by Huang et al. (31).

### Potential covariates.

Maternal prepregnancy weight and height were self-reported by mothers and used to calculate maternal overweight (BMI ≥25 kg/m^2^). Categories of maternal education included the following: *1*) Certificate of Secondary Education, vocational level, or no educational qualifications; *2*) O level (an examination taken at age 16 y, the age limit for leaving school in the United Kingdom); and *3*) A level (an examination taken at age 18 y) or university degree. Children’s Tanner stage for pubic hair growth was obtained at 13 y of age by postal questionnaire completed by the child or the parent. Tanner stage was collapsed to pubertal stages (pre = 1, early = 2–3, and late = 4–5). Because pubertal assessment may not have been obtained in parallel to attendance at the 13-y clinic, participants with questionnaires completed ≥12 mo before or after this visit had pubertal stage set to “missing.” At age 13, children reported whether in the past year they followed a diet to lose weight or keep from gaining weight with the following possible responses: always, often, several times, a couple of times, and never on a diet. This was collapsed to a dichotomous variable (no = never and yes = all other responses). Potential dietary covariates included baseline intakes of fruit juice (100% fruit juice without added sugar, g/d), fruit and vegetables (g/d), percentage of energy from fat (% fat/d), percentage of energy from protein (% protein/d), sugar-sweetened beverages (g/d), fiber (g/d), and cereal (g/d). These dietary factors were positively or negatively associated with changes in adiposity (17, 32, 33). In addition, they tend to correlate with dairy intakes as part of a healthy eating pattern (17) and are commonly consumed along with dairy during meals: for example, milk on cereal for breakfast (34). The majority of participants had covariate information available as follows: pubertal stage (*n* = 2231; 91%), maternal overweight (*n* = 1880; 77%), maternal educational attainment (*n* = 2292; 93%), and dieting at 13 y (*n* = 2135; 87%). Dummy variables were created to account for missing status and were included in all models.

### Statistical analyses.

To examine if our final sample was affected by selection bias due to missing data, we compared baseline demographic characteristics between participants retained for these analyses who had complete anthropometric, DXA, dietary, and physical activity data (*n* = 2455) with those excluded because of missing diet or physical activity (*n* = 2647) by using Student’s *t* tests and chi-square tests for sample means and proportions, respectively. We tested 3 separate dietary exposures: total and full- and reduced-fat dairy intakes. We treated these variables as quantiles at baseline (10 y). Outcomes at age 13 were excess TBFM and overweight. We additionally examined change in BMI from baseline to follow-up. Because baseline dairy intakes differed between boys and girls (336 vs. 265 g/d) and the prevalence of baseline overweight increased with higher intakes of reduced-fat dairy, dairy was categorized into sex- and BMI-specific quartiles. In sex-specific quartiles, there was a lower prevalence of overweight comparing quartiles 1 and 4 of reduced-fat dairy intakes at 10 y (14.8% vs. 23.1%, respectively); conversely, there was a higher prevalence in quartile 1 vs. quartile 4 of full-fat dairy intakes (19.2% vs.15.9%, respectively). A similar proportion of participants were overweight in quartiles 1 and 4 of total dairy intake quartiles (21.4% and 21.0%, respectively; data not shown). This suggests that overweight children consume greater amounts of reduced-fat dairy products compared with full-fat varieties, perhaps because parents of overweight children may provide reduced-fat dairy as a means to manage weight. Because BMI was correlated with dairy intakes, we created BMI-specific categories to reduce the possibility of reverse causation in our results. To develop the dairy consumption categories, sex-specific, age- and height-adjusted BMI quartiles were created and then quartiles of dairy intake were derived within each sex and BMI stratum. These same methods were used to derive categories of dairy serving intakes.

Because of the high prevalence of nonconsumption of reduced-fat dairy (as high as 36% in some strata), children in the bottom 36% of reduced-fat dairy intake were designated as the lowest category (C1) and the remaining 64% of participants were categorized by using tertiles (C2–C4). Total fat mass was not measured at age 10, when diet was assessed; therefore, we could not re-create dairy categories using TBFM as opposed to BMI. However, it is likely that deriving adiposity- and sex-specific dairy intake categories by using baseline BMI distributed similar proportions of baseline TBFM amounts across dairy consumption groups because BMI and TBFM were very strongly correlated. Correlations between TBFM at age 11 and BMI at age 11 and with BMI at age 10 were very strong: 0.94 and 0.90, respectively (data not shown).

Several sets of models using multivariable logistic regression were created to determine the effects of categorical intakes of our 3 exposure variables—total and full- and reduced-fat dairy—at age 10 on risk of excess TBFM and overweight at age 13; the lowest category of dairy consumption was set as the reference group. Full- and reduced-fat dairy were modeled together. A similar model building strategy was used for each set of exposure and outcome analyses. Model 1 (simple) adjusted for sex, dairy intake at follow-up, and age at baseline along with height and adiposity (continuous). Excess TBFM models were adjusted for baseline BMI because TBFM was not measured at age 10, as noted previously. Model 2 (demographic characteristics) included model 1 covariates plus maternal education, maternal overweight status, and physical activity, pubertal stage, and dieting at follow-up. Model 3 (diet) included model 2 factors as well as baseline intakes of cereal, total fat, total protein, fiber, 100% fruit juice, fruit and vegetables, and sugar-sweetened beverages. Model 4 (dietary reporting errors) adjusted for model 3 factors and dietary reporting errors at follow-up, and this was considered our primary model. Although total energy intake (kcal/d) may lie in the causal pathway between dietary intake and weight gain, our final model further adjusted for baseline total energy (kcal/d) to address potential confounding by differences in body size and physical activity and isolate independent effects of diet (model 5). We conducted tests for linear trend by treating the independent variable as continuous rather than categorical. The relation between categories of dairy intakes and adiposity were further explored by using change in BMI (13 y minus 10 y) as an outcome. ANCOVA (PROC GLM in SAS) was used, with a similar modeling strategy as that described for logistic regression models.

All models were adjusted for 13-y intakes of dairy. Some evidence suggests that intakes of dairy products, particularly milk, decrease during childhood and adolescence (35–37). In our sample, most participants (61%) at age 13 y were not in the same total dairy intake quartile as at age 10 y. If dairy intakes influence adiposity, adjustment for 13-y consumption would be necessary to isolate the effect of 10-y dairy intakes on 13-y risk of excess fat accumulation. In sensitivity analyses, we modeled these associations without adjustment for 13-y dairy consumption and found that the directionality and effect magnitudes were comparable to those models that adjusted for follow-up dairy intake (data not shown).

We explored whether the relations between dairy consumption and excess adiposity were influenced by sex or baseline weight status as suggested by other studies (38, 39). To test for effect modification, an interaction term for sex (e.g., sex × dairy) or baseline overweight status (e.g., sex × BMI) was included in models for each outcome. Two-sided *P* values <0.05 were considered significant. Data were analyzed by using the SAS software package (version 9.2). Values are means ± SDs unless otherwise noted.

## Results

### 

#### Baseline sample characteristics.

Participants (*n* = 2455; 53% girls) were on average 10.6 ± 0.2 y of age at baseline (10 y) and 13.8 ± 0.2 y at follow-up (13 y). The average amount of time between the baseline and follow-up visits was 3.2 ± 0.2 y. Mean BMI and the prevalence of overweight were similar across dairy-intake categories at baseline (**Table 1**). Across total and full- and reduced-fat dairy intake categories (g/d), the proportion of participants considered as plausible dietary reporters at the follow-up visit increased. Approximately 25% of children within each reduced-fat dairy intake category identified as having dieted at age 13 y, whereas the proportion of dieters decreased with increasing total and full-fat dairy intakes. The proportion of mothers obtaining an A level certificate or university degree was 5% higher among children in the top category of reduced-fat dairy intakes compared with those in the top category for full-fat dairy.

**TABLE 1:** Sample characteristics of 2455 children by categories of dairy consumption^1^

	Total dairy (g/d)				Full-fat dairy (g/d)				Reduced-fat dairy (g/d)			
Sample characteristics	Q1(*n* = 610)	Q2(*n* = 617)	Q3(*n* = 614)	Q4(*n* = 614)	Q1(*n* = 612)	Q2(*n* = 619)	Q3(*n* = 610)	Q4(*n* = 614)	C1(*n* = 873)	C2(*n* = 524)	C3(*n* = 531)	C4(*n* = 527)
Age, *y*	10.6 ± 0.2	10.6 ± 0.2	10.6 ± 0.2	10.6 ± 0.2	10.6 ± 0.2	10.6 ± 0.2	10.6 ± 0.2	10.6 ± 0.2	10.6 ± 0.2	10.6 ± 0.2	10.6 ± 0.2	10.6 ± 0.2
Girls, *%*	53.4	53.3	53.1	53.4	53.4	53	53.4	53.4	53	53.6	53.9	52.9
Height, *cm*	143 ± 7	144 ± 7	144 ± 6	145 ± 7	144 ± 6	144 ± 7	144 ± 7	144 ± 6	143 ± 6	144 ± 7	144 ± 7	144 ± 7
BMI, *kg/m^2^*	18.1 ± 2.9	18.1 ± 2.8	18.1 ± 2.9	18.2 ± 2.9	18.1 ± 2.9	18.2 ± 2.9	18.2 ± 2.9	18 ± 2.8	18 ± 2.8	18.2 ± 3	18.2 ± 2.8	18.3 ± 3
Overweight,^2^ *%*	20	20.3	20.7	21.8	20.4	22.1	20.8	19.4	19.4	20.8	21.7	21.8
Pubertal stage at 13 y,^3^ *%*												
Pre	6	5	6	5	5	5	6	6	5	6	6	6
Early	38	36	37	37	41	39	32	36	37	38	40	40
Late	56	59	58	58	54	55	63	59	58	57	54	54
Dietary reporting errors at 13 y, *%*												
Under	41	40	36	30	40	38	31	31	37	37	40	33
Plausible	40	43	43	46	40	44	45	45	41	48	42	44
Over	20	17	21	24	20	19	24	24	23	16	18	24
Dieting at 13 y,^4^ *%*	28	24	25	22	25	27	27	20	25	25	25	25
Maternal educational status, *%*												
None/CSE/vocational	20	14	15	15	19	14	15	15	18	16	13	15
O level	34	35	38	35	34	35	33	40	37	36	34	34
A level/university degree	46	51	48	50	47	51	52	46	46	48	52	51
Maternal overweight,^2^ *%*	36	36	34	34	35	34	36	35	35	38	33	35
Physical activity at 13 y,^5^ *counts/min*	530 ± 185	538 ± 178	535 ± 181	528 ± 174	535 ± 187	534 ± 178	535 ± 177	527 ± 177	532 ± 181	528 ± 173	536 ± 174	535 ± 188

1 Values are means ± SDs or percentages from baseline (age 10 y) unless otherwise indicated. Quartiles of total and full-fat dairy intake (g/d) are sex- and baseline-BMI (quartile)–specific. The lowest category (C1) of reduced-fat dairy intake (g/d) was defined as the bottom 36% of consumers because of a large number of participants with no consumption of reduced-fat dairy products at age 10 y (as much as 36% in some sex- and BMI-specific strata). The remaining participants were categorized using tertiles (C2–C4). C, category; CSE, Certificate of Secondary Education; Q, quartile.

2 Child overweight or obesity was defined by using International Obesity Taskforce age- and sex-specific weight categories (27). Mothers with a BMI ≥ 25 kg/m^2^ were categorized as overweight.

3 Tanner stage for pubic hair growth was self-reported by postal questionnaire and collapsed to pubertal stage defined as pre (Tanner = 1), early (Tanner = 2–3), and late (Tanner = 4–5).

4 Dieting status at age 13 y was self-reported by questionnaire in response to the question “During the past year, did you go on a diet to lose weight or keep from gaining weight?” (always, often, several times, a couple of times, never on diet). This variable was collapsed to a dichotomous variable (yes or no).

5 Physical activity was measured by uniaxial accelerometer in counts per minute.

Mean baseline consumption of total and full- and reduced-fat dairy in boys was 336 ± 206, 152 ± 179, and 185 ± 193 g/d, respectively, and in girls was 265 ± 182, 114 ± 138, and 151 ± 164 g/d, respectively. Milk was the largest contributor to total dairy intakes at baseline: 261 ± 194 g/d for boys and 195 ± 168 g/d for girls. Across full-fat dairy quartiles, the amount of reduced-fat dairy products consumed decreased (**Table 2**). Conversely, with increasing consumption of reduced-fat dairy products there was an accompanying decrease in full-fat dairy intakes.

**TABLE 2:** Intakes of dairy and dietary covariates among 2455 children at baseline (age 10 y) by categories of dairy consumption^1^

	Total dairy^2^ (g/d)				Full-fat dairy (g/d)				Reduced-fat dairy (g/d)			
Sample characteristics	Q1(*n* = 610)	Q2(*n* = 617)	Q3(*n* = 614)	Q4(*n* = 614)	Q1(*n* = 612)	Q2(*n* = 619)	Q3(*n* = 610)	Q4(*n* = 614)	C1(*n* = 873)	C2(*n* = 524)	C3(*n* = 531)	C4(*n* = 527)
Total dairy, *g/d*	88 ± 54	211 ± 44	330 ± 62	563 ± 155	218 ± 184	263 ± 187	301 ± 181	411 ± 183	227 ± 199	215 ± 139	290 ± 98	508 ± 165
Full-fat dairy, *g/d*	46 ± 47	100 ± 83	139 ± 125	242 ± 238	9 ± 10	51 ± 16	120 ± 39	348 ± 176	218 ± 202	109 ± 139	75 ± 86	69 ± 65
Milk	14 ± 35	50 ± 78	74 ± 117	167 ± 233	0.1 ± 1.2	4 ± 14	37 ± 56	263 ± 192	162 ± 191	53 ± 125	21 ± 68	13 ± 41
Cheese	9 ± 13	11 ± 15	14 ± 18	14 ± 17	6 ± 8	13 ± 16	15 ± 19	15 ± 19	12 ± 16	12 ± 16	13 ± 17	13 ± 17
Yogurt	10 ± 23	18 ± 33	25 ± 40	33 ± 52	0.8 ± 5	16 ± 22	32 ± 40	38 ± 57	20 ± 38	23 ± 40	21 ± 38	23 ± 43
Dairy desserts	13 ± 24	21 ± 32	26 ± 38	28 ± 38	2.4 ± 7	18 ± 22	35 ± 38	32 ± 44	24 ± 37	22 ± 33	22 ± 34	20 ± 31
Reduced-fat dairy, *g/d*	42 ± 50	111 ± 83	191 ± 125	321 ± 246	209 ± 182	212 ± 186	182 ± 181	63 ± 115	9 ± 20	106 ± 38	215 ± 49	439 ± 154
Milk	35 ± 47	98 ± 81	169 ± 123	296 ± 243	189 ± 174	196 ± 181	165 ± 176	49 ± 111	5 ± 16	85 ± 50	194 ± 60	409 ± 160
Cheese	0.3 ± 3.0	0.2 ± 2.4	0.5 ± 3.8	0.6 ± 3.6	0.6 ± 4.3	0.5 ± 3.8	0.3 ± 2.3	0.2 ± 2	0.1 ± 1.4	0.5 ± 3.7	0.5 ± 3.9	0.6 ± 4
Yogurt	5 ± 18	10 ± 24	18 ± 39	21 ± 43	17 ± 41	12 ± 28	13 ± 32	12 ± 31	3 ± 12	17 ± 34	17 ± 35	24 ± 47
Dairy desserts	2 ± 9	3 ± 13	3 ± 12	4 ± 15	3 ± 13	4 ± 13	4 ± 14	1 ± 9	1 ± 6	4 ± 13	3 ± 13	6 ± 18
Total fat, *% of energy/d*	37 ± 5	36 ± 5	36 ± 4	36 ± 5	35 ± 5	36 ± 5	37 ± 5	37 ± 4	38 ± 5	36 ± 5	36 ± 4	35 ± 5
Total protein, *% of energy/d*	13 ± 3	13 ± 2	13 ± 2	14 ± 2	14 ± 2	14 ± 2	13 ± 2	13 ± 2	13 ± 2	13 ± 2	13 ± 2	14 ± 2
Fruit and vegetables, *g/d*	129 ± 105	136 ± 102	135 ± 93	150 ± 102	138 ± 115	137 ± 94	141 ± 100	134 ± 95	124 ± 97	147 ± 107	136 ± 99	151 ± 100
Fruit juice, *g/d*	133 ± 167	125 ± 155	129 ± 149	122 ± 154	124 ± 149	134 ± 164	124 ± 154	127 ± 158	127 ± 165	132 ± 156	125 ± 149	125 ± 148
Sugar-sweetened beverages, *g/d*	168 ± 211	123 ± 174	120 ± 162	113 ± 164	139 ± 196	126 ± 178	129 ± 171	131 ± 174	161 ± 200	117 ± 166	123 ± 171	103 ± 159
Fiber, *g/d*	11 ± 4	12 ± 3	12 ± 3	12 ± 4	12 ± 4	12 ± 3	12 ± 3	12 ± 4	11 ± 4	12 ± 4	12 ± 3	12 ± 4
Cereal, *g/d*	15 ± 27	28 ± 23	34 ± 23	35 ± 24	27 ± 31	26 ± 23	26 ± 22	32 ± 25	23 ± 28	25 ± 21	33 ± 23	35 ± 24
Dietary calcium, *mg/d*	564 ± 181	711 ± 159	864 ± 183	1140 ± 240	705 ± 273	783 ± 274	840 ± 278	955 ± 271	723 ± 286	727 ± 230	815 ± 200	1080 ± 261
Total energy, *kcal/d*	1750 ± 356	1820 ± 346	1900 ± 342	2050 ± 338	1770 ± 360	1840 ± 335	1920 ± 343	1990 ± 373	1870 ± 382	1820 ± 353	1860 ± 343	1970 ± 342
Total dairy, *%*												
Q1	—	—	—	—	43	34	22	1	44	41	2	0
Q2	—	—	—	—	24	25	28	23	23	38	41	0
Q3	—	—	—	—	18	23	27	32	16	12	49	29
Q4	—	—	—	—	15	18	22	45	17	9	9	71
Full-fat dairy, *%*												
Q1	43	24	18	15	—	—	—	—	16	27	32	32
Q2	34	25	24	18	—	—	—	—	14	30	31	33
Q3	22	28	27	22	—	—	—	—	22	25	28	27
Q4	1	22	32	45	—	—	—	—	49	19	10	8
Reduced-fat dairy, *%*												
C1	64	32	23	24	22	20	31	69	—	—	—	—
C2	35	32	10	7	23	25	22	16	—	—	—	—
C3	2	35	42	7	28	26	24	8	—	—	—	—
C4	0	0	25	61	28	28	24	7	—	—	—	—

1 Values are means ± SDs or frequencies (%) from baseline (age 10 y) unless otherwise indicated. Intakes are the mean of 3-d food records obtained at ∼10 and 13 y of age. Quartiles of total and full-fat dairy intakes (g/d) are sex- and baseline-BMI–specific. The lowest category (C1) of reduced-fat dairy intake (g/d) was defined as the bottom 36% of consumers because of a large number of participants with no consumption of reduced-fat dairy products at age 10 y (as much as 36% in some sex- and BMI-specific strata). The remaining participants were categorized using tertiles (C2–C4). Consistent with the USDA, a serving of dairy was defined as a calcium equivalent to 1 cup of milk (21). C, category; Q, quartile.

2 Dairy included milk, cheese, yogurt, and dairy-based desserts (e.g., ice cream). Dairy from mixed dishes (e.g., cheese on pizza) was not included.

#### Total dairy intakes and excess adiposity.

In the simple adjusted model (model 1), children in the top quartile of total dairy intakes (g/d) relative to those in the bottom quartile tended to have a 33% lower risk of excess fat mass (*P*-trend = 0.10) (**Table 3**). In the fully adjusted model, the relation was attenuated (*P*-trend = 0.30; model 3) and remained similar in our primary model that additionally adjusted for dietary reporting biases (*P*-trend = 0.28; model 4). The risks of overweight at age 13 y in children in the top vs. lowest intake quartiles across the 4 models were similar in magnitudes to those for excess fat mass (Table 3). In analyses using categories of servings of dairy as the exposure, we found that parameter estimates were similar in direction to those using grams of dairy (**Supplemental Table 1**). Effect estimates of excess TBFM did vary in size between dairy weight and dairy serving analyses; however, examination of the highest vs. lowest total dairy consumers showed similar results (Supplemental Table 1).

**TABLE 3:** ORs (95% CIs) for total dairy intakes at age 10 y and risk of overweight and excess fat mass at age 13 y^1^

	Total dairy intakes at 10 y^2^				
Excess adiposity at age 13 y	Q1: 88 ± 54 (0–177) g/d (*n* = 610)	Q2: 211 ± 44 (143–287) g/d (*n* = 617)	Q3: 330 ± 62 (233–439) g/d (*n* = 614)	Q4: 563 ± 155 (378–868) g/d (*n* = 614)	*P*-trend
Excess fat mass, *n (%)*	136 (22)	121 (20)	124 (20)	110 (18)	
Model 1	1.00	0.81 (0.55, 1.19)	0.88 (0.60, 1.31)	0.67 (0.44, 1.01)	0.10
Model 2	1.00	0.89 (0.60, 1.32)	0.97 (0.64, 1.45)	0.72 (0.46, 1.10)	0.19
Model 3	1.00	0.90 (0.59, 1.35)	0.98 (0.64, 1.50)	0.75 (0.47, 1.19)	0.30
Model 4	1.00	0.84 (0.56, 1.28)	0.94 (0.61, 1.45)	0.73 (0.46, 1.16)	0.28
Model 5	1.00	0.82 (0.54, 1.24)	0.88 (0.56, 1.36)	0.67 (0.41, 1.10)	0.18
Overweight, *n (%)*	124 (20)	119 (19)	126 (21)	114 (19)	
Model 1	1.00	0.92 (0.60, 1.42)	1.07 (0.69, 1.66)	0.69 (0.43, 1.10)	0.19
Model 2	1.00	0.99 (0.64, 1.53)	1.15 (0.73, 1.80)	0.73 (0.45, 1.18)	0.29
Model 3	1.00	0.92 (0.58, 1.45)	1.07 (0.67, 1.73)	0.69 (0.42, 1.16)	0.25
Model 4	1.00	0.89 (0.56, 1.41)	1.04 (0.65, 1.69)	0.69 (0.41, 1.15)	0.24
Model 5	1.00	0.85 (0.53, 1.34)	0.91 (0.56, 1.49)	0.56 (0.32, 0.97)	0.07

1 Quartiles of total dairy intakes are sex- and baseline-BMI–specific. Excess fat mass was defined as the top quintile for sex-specific and age- and height-adjusted total body fat mass (kg) and overweight was defined by using International Obesity Taskforce age- and sex-specific weight categories (27). Relations between total dairy intakes and excess adiposity were examined by multivariable logistic regression (PROC logistic in SAS). *P*-trend was determined by treating quartiles of dairy intake as a continuous variable in regression models. Model 1 (simple): age 10 y, sex, height at 10 y, total dairy at 13 y (categorical), and adiposity at 10 y (continuous); model 2 (demographic characteristics): model 1 plus maternal education and overweight status, physical activity at 13 y, pubertal stage at 13 y, and dieting at 13 y; model 3 (diet): model 2 plus age-10-y intakes of fruit juice, fruit and vegetables, total fat, total protein, sugar-sweetened beverages, fiber, and cereal; model 4 (reporting errors): model 3 additionally adjusted for dietary reporting errors at 13 y; model 5 (energy adjusted): model 4 plus adjustment for total dairy intakes. Q, quartile.

2 Values represent mean ± SD (5th–95th percentile) total dairy intakes at age 10 y.

#### Full- and reduced-fat dairy intakes and excess adiposity.

Children in the highest vs. lowest quartile of full-fat dairy intakes (g/d) had a 37% lower risk of excess fat mass at age 13 y in the simple adjusted model (model 1; *P*-trend = 0.03) (**Table 4**). Results remained consistent in fully adjusted (*P*-trend = 0.06; model 3) and primary (*P*-trend = 0.04; model 4) models. Analyses of dairy servings were also related to a reduced risk of excess TBFM, although effect sizes were attenuated by comparison, particularly among children consuming the highest vs. the lowest number of servings of full-fat dairy (*P*-trend = 0.17; model 4) (**Supplemental Table 2**).

**TABLE 4:** ORs (95% CIs) for full-fat dairy intakes at age 10 y and excess adiposity at 13 y^1^

	Full-fat dairy intakes at 10 y^2^				
Excess adiposity at age 13 y	Q1: 9 ± 10 (0–26) g/d (*n* = 612)	Q2: 50 ± 16 (27–78) g/d (*n* = 619)	Q3: 120 ± 38 (71–195) g/d (*n* = 610)	Q4: 348 ± 176 (154–709) g/d (*n* = 614)	*P*-trend
Excess fat mass, *n (%)*	134 (22)	127 (21)	120 (20)	110 (18)	
Model 1	1.00	0.84 (0.58, 1.23)	0.75 (0.51, 1.10)	0.63 (0.41, 0.95)	0.03
Model 2	1.00	0.82 (0.55, 1.20)	0.75 (0.51, 1.12)	0.64 (0.42, 0.99)	0.04
Model 3	1.00	0.82 (0.55, 1.21)	0.75 (0.50, 1.12)	0.67 (0.43, 1.04)	0.06
Model 4	1.00	0.80 (0.54, 1.19)	0.72 (0.48, 1.07)	0.64 (0.41, 1.00)	0.04
Model 5	1.00	0.78 (0.53, 1.15)	0.70 (0.47, 1.05)	0.59 (0.37, 0.94)	0.02
Overweight, *n (%)*	128 (21)	120 (19)	124 (20)	111 (18)	
Model 1	1.00	0.77 (0.50, 1.17)	0.87 (0.57, 1.34)	0.67 (0.42, 1.07)	0.17
Model 2	1.00	0.76 (0.49, 1.17)	0.86 (0.56, 1.32)	0.68 (0.42, 1.09)	0.18
Model 3	1.00	0.76 (0.49, 1.17)	0.85 (0.55, 1.32)	0.66 (0.41, 1.08)	0.21
Model 4	1.00	0.75 (0.48, 1.16)	0.83 (0.54, 1.30)	0.65 (0.40, 1.06)	0.19
Model 5	1.00	0.71 (0.46, 1.10)	0.78 (0.50, 1.22)	0.57 (0.34, 0.94)	0.06

1 Quartiles of total dairy intakes are sex- and baseline-BMI–specific. Excess fat mass was defined as the top quintile for sex-specific and age- and height-adjusted total body fat mass (kg) and overweight was defined by using International Obesity Taskforce age- and sex-specific weight categories (27). Relations between total dairy intakes and excess adiposity were examined by multivariable logistic regression (PROC logistic in SAS). *P*-trend was determined by treating quartiles of dairy intake as a continuous variable in regression models. Model 1 (simple): age 10 y, sex, height at 10 y, total dairy at 13 y (categorical), and adiposity at 10 y (continuous); model 2 (demographic characteristics): model 1 plus maternal education and overweight status, physical activity at 13 y, pubertal stage at 13 y, and dieting at 13 y; model 3 (diet): model 2 plus age-10-y intakes of fruit juice, fruit and vegetables, total fat, total protein, sugar-sweetened beverages, fiber, and cereal; model 4 (reporting errors): model 3 additionally adjusted for dietary reporting errors at 13 y; model 5 (energy adjusted): model 4 plus adjustment for total dairy intakes. Q, quartile.

2 Values represent mean ± SD (5th–95th percentile) full-fat dairy intakes at age 10 y.

Associations between reduced-fat dairy intakes and risk of excess TBFM were attenuated compared with those for full-fat dairy. For example, children consuming the highest vs. lowest amounts of reduced-fat dairy products (g/d) showed a 28% reduced risk of excess TBFM, but this estimate did not attain significance (*P*-trend = 0.23; model 4) (**Table 5**). In analyses of dairy servings, no relation was evident (*P*-trend = 0.65; risk of excess TBFM, model 4) (**Supplemental Table 3**).

**TABLE 5:** ORs (95% CIs) of reduced-fat dairy intakes at age 10 y and excess adiposity at 13 y^1^

	Reduced-fat dairy intakes at 10 y^2^				
Excess adiposity at age 13 y	C1: 9 ± 20 (0–50) g/d (*n* = 873)	C2: 106 ± 38 (42–166) g/d (*n* = 524)	C3: 215 ± 49 (148–300) g/d (*n* = 531)	C4: 439 ± 154 (265–769) g/d (*n* = 527)	*P-*trend
Excess fat mass, *n (%)*	179 (21)	107 (20)	103 (19)	102 (19)	
Model 1	1.00	0.86 (0.58, 1.27)	0.78 (0.52, 1.17)	0.75 (0.48, 1.16)	0.16
Model 2	1.00	0.84 (0.56, 1.26)	0.82 (0.54, 1.25)	0.72 (0.46, 1.13)	0.16
Model 3	1.00	0.87 (0.58, 1.31)	0.82 (0.53, 1.27)	0.74 (0.46, 1.20)	0.19
Model 4	1.00	0.85 (0.56, 1.29)	0.79 (0.51, 1.23)	0.77 (0.47, 1.25)	0.23
Model 5	1.00	0.85 (0.56, 1.28)	0.76 (0.49, 1.19)	0.72 (0.44, 1.19)	0.15
Overweight, *n (%)*	172 (20)	103 (20)	100 (19)	108 (20)	
Model 1	1.00	0.87 (0.56, 1.34)	0.75 (0.48, 1.18)	0.87 (0.54, 1.41)	0.44
Model 2	1.00	0.85 (0.55, 1.33)	0.77 (0.49, 1.22)	0.86 (0.53, 1.40)	0.45
Model 3	1.00	0.85 (0.54, 1.33)	0.72 (0.44, 1.16)	0.83 (0.50, 1.40)	0.39
Model 4	1.00	0.85 (0.54, 1.33)	0.71 (0.44, 1.15)	0.85 (0.50, 1.44)	0.42
Model 5	1.00	0.83 (0.53, 1.30)	0.67 (0.41, 1.09)	0.74 (0.43, 1.28)	0.19

1 The lowest category (C1) of reduced-fat dairy intake was defined as the bottom 36% of consumers because of a large number of participants with no consumption of reduced-fat dairy products at age 10 y (as much as 36% in some sex- and BMI-specific strata). The remaining participants were categorized using tertiles (C2–C4). Excess fat mass was defined as the top quintile for sex-specific and age- and height-adjusted total body fat mass (kg) and overweight was defined by using International Obesity Taskforce age- and sex-specific weight categories (27). Multivariable logistic regression (PROC logistic in SAS) was used to examine the effects of dairy intake on the odds of excess adiposity. *P*-trend was determined by treating amounts of intake as a continuous variable. Model 1 (simple): age 10 y, sex, height at 10 y, total dairy at 13 y (categorical), and adiposity at 10 y (continuous); model 2 (demographic characteristics): model 1 plus maternal education and overweight status, physical activity at 13 y, pubertal stage at 13 y, and dieting at 13 y; model 3 (diet): model 2 plus age-10-y intakes of fruit juice, fruit and vegetables, total fat, total protein, sugar-sweetened beverages, fiber, and cereal; model 4 (reporting errors): model 3 additionally adjusted for dietary reporting errors at 13 y; model 5 (energy adjusted): model 4 plus adjustment for total dairy intakes. C, category.

2 Values represent mean ± SD (5th–95th percentile) reduced-fat dairy intakes at age 10 y.

#### Dairy intakes and change in BMI.

In models 1 and 4, those with the highest intakes of total dairy tended (*P* < 0.1) to have smaller gains in BMI (kg/m^2^) from ages 10 to 13 y compared with those with the lowest intakes of dairy (data not shown). There was a significant linear trend in model 4 (*P* = 0.04) for smaller gains in BMI among children with higher intakes of total dairy. With regard to full-fat dairy products, those children with the highest intakes compared with those with the lowest intakes had smaller gains in BMI during follow-up in models 1 to 4 (*P* < 0.05 for quartile 1 vs. quartile 4; *P*-trend < 0.01) (**Table 6**). In similar sets of analyses, a relation between reduced-fat dairy product intakes and changes in BMI was not detected.

**TABLE 6:** Relation between full-fat dairy intakes at age 10 y and change in BMI from ages 10 to 13 y^1^

	Full-fat dairy intakes at 10 y^2^				
	Q1: 9 ± 10 (0–26) g/d (*n* = 612)	Q2: 50 ± 16 (27–78) g/d (*n* = 619)	Q3: 120 ± 38 (71–195) g/d (*n* = 610)	Q4: 348 ± 176 (154–709) g/d (*n* = 614)	*P*-trend
	*ΔBMI/3 y*			
Model 1	2.3 (2.2, 2.4)	2.2 (2.0, 2.3)	2.1 (2.0, 2.3)	2.0 (1.8, 2.2)*	0.005
Model 2	2.8 (2.5, 3.0)	2.7 (2.4, 2.9)	2.6 (2.3, 2.8)	2.5 (2.2, 2.7)*	0.004
Model 3	2.8 (2.5, 3.0)	2.7 (2.4, 2.9)	2.6 (2.3, 2.8)	2.5 (2.2, 2.7)*	0.004
Model 4	2.8 (2.5, 3.0)	2.6 (2.4, 2.9)	2.6 (2.3, 2.8)	2.5 (2.2, 2.7)*	0.004
Model 5	2.7 (2.5, 3.0)	2.6 (2.4, 2.9)	2.6 (2.3, 2.8)	2.5 (2.2, 2.7)^#x2020^	0.009

1 Values represent mean (95% CI) changes. Quartiles of full-fat dairy intakes are sex- and baseline-BMI–specific. Relations between total dairy intakes and changes in BMI were examined by ANCOVA (PROC GLM in SAS). *P*-trend was determined by treating quartiles of dairy intake as a continuous variable in multivariable models. Model 1 (simple): age 10 y, sex, height at 10 y, total dairy at 13 y (categorical), and adiposity at 10 y (continuous); model 2 (demographic characteristics): model 1 plus maternal education and overweight status, physical activity at 13 y, pubertal stage at 13 y, and dieting at 13 y; model 3 (diet): model 2 plus age-10-y intakes of fruit juice, fruit and vegetables, total fat, total protein, sugar-sweetened beverages, fiber, and cereal; model 4 (reporting errors): model 3 additionally adjusted for dietary reporting errors at 13 y; model 5 (energy adjusted): model 4 plus adjustment for total dairy intakes. *P < 0.05 and ^†^*P* = 0.05 compared with Q1 full-fat dairy intakes. Q, quartile.

2 Values represent mean ± SD (5th–95th percentile) full-fat dairy intakes at age 10 y.

#### Effect modification and total energy.

There was no evidence that the relation between grams of dairy consumption and risk of excess adiposity differed by sex or baseline overweight status (*P* > 0.1 for all). In general, adjustment for total energy intake (model 5) did not change the interpretation of our results

## Discussion

Total and full- and reduced-fat dairy consumption during preadolescence was not associated with excess fat accumulation during early adolescence. In general, although our estimates did not reach significance in many cases, they are consistent with the inverse relations between dairy intakes and risk of excess adiposity shown in other studies, particularly for full-fat dairy products and risk of excess TBFM. Children in the highest vs. the lowest quartile of full-fat dairy intakes (g/d) at age 10 y had a >30% reduced risk of excess body fat mass and overweight at age 13 y. Moreover, higher intakes of full-fat dairy products were related to a lower gain in BMI during follow-up. Children in the top quartile consumed, on average, 348 g/d (1.6 servings/d) of full-fat dairy products at age 10 y, whereas those in the bottom quartile consumed ∼9 g/d (0.1 serving/d). Collectively, these data suggest a protective effect of dairy consumption but should be interpreted with caution given the wide confidence limits of our estimates.

The evidence linking total dairy consumption and adiposity in children or adolescents is equivocal with observational studies that report inverse (22, 23, 40–42), positive (14), and null (41–45) associations. Only our study and a few others (22, 23, 44, 45) were prospectively designed, which is critical to minimize the potential effect of reverse causation (i.e., parents providing dairy products to their child to manage their child’s weight). Yet, some of these prospective studies found that total dairy consumption was not positively related to weight gain (44, 45), whereas others reported a weight-reducing effect (22, 23). The method of dietary assessment could partly explain these differing results. Those that reported an inverse relation used 3-d food records (the method used in our study), whereas others used an FFQ (44, 45). Diet records are not as dependent on the participant’s memory, and multiple days reduce within-person variability; this method is thus less prone to dietary reporting errors that can result in misclassification (46). In our study, effect estimates of total dairy intake on excess TBFM or overweight consistently indicated an inverse association, but all estimates had wide confidence limits and tests of linear trend were not significant (*P* = 0.07–0.30). Therefore, our data in conjunction with previous findings indicate that dairy intakes do not have an adverse effect on childhood adiposity and may have a small protective effect.

Compared with the number of studies of total dairy intake and adiposity in children, those examining associations by dairy fat amount are scarce (44, 47, 48). Berz et al. (47) found that 9-y-old girls consuming ≥2.5 servings/d of low-fat dairy experienced smaller gains in BMI over a 10-y period compared with those consuming <1 serving/d and 1 to <2.25 servings/d. Unfortunately, results with full-fat dairy were not reported. Although not specifically examining total dairy intake, a few studies of full-fat milk found inverse relations with adiposity (45, 49). Children (ages 3–11 y) participating in a cross-sectional study had lower BMI Z-scores with higher full-fat milk consumption (49). This is consistent with a longitudinal analysis in which full-fat milk intake at age 2 y was negatively associated with BMI Z-score at age 3 y in full-cohort analyses (45), although reduced-fat milk consumption was not. In that study, baseline milk intake was not related to incident overweight, but the reduction in sample size for these analyses may explain the null finding. In another study conducted in ALSPAC, the authors found that full-fat milk consumption at age 10 y, as well as total intakes and intakes of reduced-fat varieties, were not associated with body fat at age 13 y (50).

In contrast to our data and those of others, Phillips et al. (44) did not detect any associations between full- or low-fat dairy, or total intakes, and BMI Z-score and percentage of body fat by bioelectrical impedance among 8- to 12-y-old girls followed until 4 y postmenarche. Methodologic differences in adiposity measurement or dietary assessment methods as noted earlier could result in these findings. Notably, our study used the gold-standard DXA to assess fat mass, whereas bioelectrical impedance was shown to overestimate percentage of fat in lean persons while underestimating fat percentage in those who are obese (51). In a recent cross-sectional study conducted in 3- to 5-y-old U.S. Head Start participants, low-fat dairy consumption was positively related to BMI Z-score and increased odds of obesity (48), but these findings could be explained by reverse causation as previously explained. In general, the paucity of evidence and the heterogeneity in research methods between studies make it difficult to draw definitive conclusions regarding the effects of full- or reduced-fat dairy intake on childhood adiposity (15–17). Thus, our study contributes to the literature through its rigorous analysis of dairy based on fat content and excess adiposity in children with its use of a prospective design, its accounting for reporting error biases, and the use of precise measures of adiposity (DXA).

There are a number of potential mechanisms by which dairy intakes may influence adiposity. Dietary calcium (e.g., through dairy) can upregulate adipocyte lipolysis (52), inhibit lipogenesis (52), and reduce fat absorption (13). In addition, dairy-specific proteins can increase satiety signaling (10). In our study, high full-fat dairy intake was more protective against excess weight and limited gain in BMI, whereas reduced-fat dairy intake was unrelated to these outcomes. Dairy fat is a source of medium-chain TGs (12) and CLA (25), both of which were associated with weight change. For example, medium-chain TGs may stimulate diet-induced thermogenesis in animals and humans (26) and CLA in animals was shown to promote weight reduction (25).

It is important to note that the 10-y total dairy intakes of our study participants would be considered suboptimal (1.4 servings/d) compared with the current U.S. dietary recommendation of 3 servings/d for children ages 9 to 18 y (53). Moreover, consumption of mostly fat-free and low-fat dairy products is encouraged. Despite low intakes, our data suggest that full-fat dairy may potentially be protective against excess weight gain. More research is needed to reproduce these results in children, but our findings are consistent with the adult literature (54). In a recent systematic review, Kratz et al. (54) found that most adult observational studies (11 of 16) reported that higher full-fat dairy consumption (vs. lower) relates to smaller body size and lower body weight.

Our study has a number of noteworthy strengths. The prospective design reduced the chance of reverse causation, although this may still be an issue due to the directly observable nature of weight gain. We adjusted for many covariates, including objectively measured physical activity and dieting status, but residual confounding still remains a possibility. Repeated measures of diet obtained by the gold standard, 3-d dietary records, and the use of DXA provided valid assessments of diet and adiposity. In contrast to most studies of dairy and obesity, we accounted for dietary reporting errors, a bias that can obfuscate diet-adiposity associations (31).

Several limitations should also be considered. Total dairy intakes were underestimated because dairy from mixed dishes such as cheese on pizza were excluded. However, this likely resulted in a minimal degree of exposure misclassification because milk contributes the most to total dairy intakes of European children (55). Total dairy intakes in this cohort of British children were relatively small, especially in comparison to those of U.S. children (41). Dairy may have a greater public health impact on adiposity in groups in whom consumption is higher. Misclassification could have occurred because we determined dietary misreporting status at age 13 vs. age 10. Energy reporting accuracy may decline as children age (56), perhaps explaining why parameter estimates were similar after adjustment for dietary misreporting. Alternatively, dairy foods could be reported with greater accuracy because of their promoted health benefits (socially desirable) (57) and consumption at main meals (e.g., milk on cereal) (34). As noted previously, most of our results are not significant. The issue of the importance of statistical significance is controversial, explained in part to the arbitrary value that is considered to be significant (58, 59). However, in light of the consistency of an inverse association observed in our study as well as in others, further research is warranted using similar robust methods to reproduce these findings.

In conclusion, our data indicate that higher dairy consumption during preadolescence does not adversely affect excess fat deposition during early adolescence. Most associations between dairy intakes and excess adiposity were inverse, particularly for full-fat dairy products, suggesting a protective relation. However, given the wide confidence limits of our parameter estimates, additional prospective research is warranted to examine the relation between dairy intake and obesity.

## Supplementary Material

Online Supporting Material
