# Supplementary material for: Dairy Intakes at Age 10 Years Do Not Adversely Affect Risk of Excess Adiposity at 13 Years
Source: J Nutr. 2014 Apr 17;144(7):1081–90. doi: 10.3945/jn.113.183640 (PMC4056647; doi:10.3945/jn.113.183640)
Supplement: Online Supporting Material [file supp_144_7_1081__index.html]

Online Supporting Material 

# Dairy Intakes at Age 10 Years Do Not Adversely Affect Risk of Excess Adiposity at 13 Years

## Online Supporting Material

**Files in this Data Supplement:**

- Online Supporting Material - Tables 1-3
