# Supplementary material for: Dairy Intakes at Age 10 Years Do Not Adversely Affect Risk of Excess Adiposity at 13 Years
Source: J Nutr. 2014 Apr 17;144(7):1081–90. doi: 10.3945/jn.113.183640 (PMC4056647; doi:10.3945/jn.113.183640)
Supplement: Online Supporting Material [file jn.113.183640_nutrition183640SupplementaryData1.docx]

| **Supplemental Table 1.** Odds ratios (ORs) and 95% confidence intervals (CIs) of total dairy intakes (s/d) at age 10 y and risk of overweight and excess fat mass at age 13 y *^1^* | | | | | | |
| --- | --- | --- | --- | --- | --- | --- |
|  |  | Total dairy intakes at 10 y, s/d | | | | *P* for trend |
|  |  | Q1 | Q2 | Q3 | Q4 |  |
| Excess adiposity at age 13 y |  | 0.44 ± 0.25  (0 - 0.83) *^2^* | 1.03 ± 0.19  (0.72 - 1.36) | 1.57 ± 0.25  (1.17 - 2.02) | 2.59 ± 0.64  (1.81 - 3.81) |  |
|  |  | *n*=609 | *n*=616 | *n*=616 | *n*=614 |  |
| Excess fat mass | *n* [%] | 142 [23] | 119 [19] | 112 [18] | 118 [19] |  |
|  |  |  | OR (95% CI) | OR (95% CI) | OR (95% CI) |  |
| Model 1 |  | 1.00 | 0.69 (0.48, 1.01) | 0.59 (0.40, 0.88) | 0.67 (0.45, 1.00) | 0.03 |
| Model 2 |  | 1.00 | 0.77 (0.52, 1.14) | 0.64 (0.43, 0.96) | 0.72 (0.48, 1.09) | 0.08 |
| Model 3 |  | 1.00 | 0.78 (0.52, 1.16) | 0.67 (0.44, 1.02) | 0.77 (0.50, 1.19) | 0.19 |
| Model 4 |  | 1.00 | 0.78 (0.52, 1.18) | 0.69 (0.45, 1.06) | 0.78 (0.50, 1.21) | 0.24 |
| Model 5 |  | 1.00 | 0.75 (0.50, 1.13) | 0.66 (0.43, 1.01) | 0.73 (0.46, 1.18) | 0.16 |
| Overweight | *n* [%] | 130 [21] | 124 [20] | 112 [18] | 117 [19] |  |
|  |  |  | OR (95% CI) | OR (95% CI) | OR (95% CI) |  |
| Model 1 |  | 1.00 | 0.92 (0.61, 1.40) | 0.66 (0.42, 1.01) | 0.69 (0.44, 1.08) | 0.04 |
| Model 2 |  | 1.00 | 1.01 (0.66, 1.55) | 0.70 (0.45, 1.09) | 0.73 (0.46, 1.16) | 0.08 |
| Model 3 |  | 1.00 | 0.97 (0.63, 1.51) | 0.68 (0.43, 1.08) | 0.72 (0.44, 1.16) | 0.08 |
| Model 4 |  | 1.00 | 1.00 (0.64, 1.56) | 0.70 (0.44, 1.11) | 0.73 (0.45, 1.19) | 0.10 |
| Model 5 |  | 1.00 | 0.92 (0.59, 1.44) | 0.62 (0.38, 0.99) | 0.59 (0.35, 0.99) | 0.02 |
| *^1^* Consistent with the USDA, a serving of dairy was defined as a calcium equivalent to 1 cup of milk (21). Quartiles of servings per day (s/d) of total dairy intakes are sex- and baseline BMI-specific. Excess fat mass was defined as the top quintile for sex-specific and age- and height-adjusted total body fat mass (kg) and overweight using International Obesity Taskforce age- and sex-specific weight categories (27). Relationships between total dairy intakes and excess adiposity were examined by multivariable logistic regression (proc logistic in SAS). *P* for trend was determined by treating quartiles of dairy intake as a continuous variable in regression models. **Model 1** (simple) - age 10 y, sex, height 10 y, total dairy 13 y (categorical), and adiposity 10 y (continuous); **Model 2** (demographics) – model 1 plus maternal education and overweight status, physical activity13 y, pubertal stage13 y, and dieting 13 y; **Model 3** (diet) – model 2 plus 10 y intakes of fruit juice, fruits and vegetables, total fat, total protein, sugar-sweetened beverages, fiber, and cereal; **Model 4** (reporting errors) – model 3 additionally adjusted for dietary reporting errors 13 y. **Model 5** (energy adjusted) – model 4 plus adjustment for total dairy intakes.  ^2^ Values represent the mean ± SD and (5^th^ - 95^th^ percentiles) of total dairy intakes at age 10 y in s/d. | | | | | | |

| **Supplemental Table 2.** Odds ratios (ORs) and 95% confidence intervals (CIs) of full-fat dairy intakes (s/d) at age 10 y and excess adiposity at 13 y *^1^* | | | | | | |
| --- | --- | --- | --- | --- | --- | --- |
|  |  | Full-fat dairy intakes at 10 y, s/d | | | | *P* for trend |
|  |  | Q1 | Q2 | Q3 | Q4 |  |
| Excess adiposity at age 13 y |  | 0.07 ± 0.08 (0 - 0.21) *^2^* | 0.36 ± 0.10  (0.22 - 0.52) | 0.76 ± 0.17  (0.51 - 1.07) | 1.72 ± 0.67  (1.00 - 3.03) |  |
|  |  | *n*=615 | *n*=610 | *n*=616 | *n*=614 |  |
| Excess fat mass | *n* [%] | 140 [23] | 122 [20] | 115 [19] | 114 [23] |  |
|  |  |  | OR (95% CI) | OR (95% CI) | OR (95% CI) |  |
| Model 1 |  | 1.00 | 0.72 (0.49, 1.05) | 0.64 (0.44, 0.94) | 0.65 (0.43, 0.97) | 0.03 |
| Model 2 |  | 1.00 | 0.74 (0.50, 1.10) | 0.65 (0.44, 0.97) | 0.70 (0.46, 1.07) | 0.08 |
| Model 3 |  | 1.00 | 0.76 (0.51, 1.12) | 0.68 (0.45, 1.01) | 0.76 (0.49, 1.17) | 0.17 |
| Model 4 |  | 1.00 | 0.73 (0.49, 1.09) | 0.66 (0.44, 0.98) | 0.75 (0.48, 1.16) | 0.17 |
| Model 5 |  | 1.00 | 0.73 (0.49, 1.09) | 0.65 (0.44, 0.98) | 0.73 (0.47, 1.15) | 0.13 |
| Overweight | *n* [%] | 129 [21] | 121 [20] | 123 [20] | 110 [18] |  |
|  |  |  | OR (95% CI) | OR (95% CI) | OR (95% CI) |  |
| Model 1 |  | 1.00 | 0.80 (0.52, 1.23) | 0.91 (0.60, 1.39) | 0.69 (0.44, 1.10) | 0.20 |
| Model 2 |  | 1.00 | 0.83 (0.53, 1.28) | 0.92 (0.60, 1.42) | 0.73 (0.46, 1.17) | 0.29 |
| Model 3 |  | 1.00 | 0.83 (0.54, 1.29) | 0.94 (0.61, 1.45) | 0.76 (0.47, 1.23) | 0.39 |
| Model 4 |  | 1.00 | 0.82 (0.53, 1.27) | 0.93 (0.60, 1.44) | 0.76 (0.47, 1.23) | 0.39 |
| Model 5 |  | 1.00 | 0.81 (0.52, 1.26) | 0.89 (0.58, 1.39) | 0.68 (0.42, 1.12) | 0.21 |
| *^1^* Consistent with the USDA, a serving of dairy was defined as a calcium equivalent to 1 cup of milk (21). Quartiles of servings per day (s/d) of total dairy intakes are sex- and baseline BMI-specific. Excess fat mass was defined as the top quintile for sex-specific and age- and height-adjusted total body fat mass (kg) and overweight using International Obesity Taskforce age- and sex-specific weight categories (27). Relationships between total dairy intakes and excess adiposity were examined by multivariable logistic regression (proc logistic in SAS). *P* for trend was determined by treating quartiles of dairy intake as a continuous variable in regression models. **Model 1** (simple) - age 10 y, sex, height 10 y, total dairy 13 y (categorical), and adiposity 10 y (continuous); **Model 2** (demographics) – model 1 plus maternal education and overweight status, physical activity13 y, pubertal stage13 y, and dieting 13 y; **Model 3** (diet) – model 2 plus 10 y intakes of fruit juice, fruits and vegetables, total fat, total protein, sugar-sweetened beverages, fiber, and cereal; **Model 4** (reporting errors) – model 3 additionally adjusted for dietary reporting errors 13 y. **Model 5** (energy adjusted) – model 4 plus adjustment for total dairy intakes.  *^2^* Values represent the mean ± SD and (5^th^ - 95^th^ percentiles) of full-fat dairy intakes at age 10 y in s/d. | | | | | | |

| **Supplemental Table 3.**  Odds ratio (ORs) and 95% confidence intervals (CIs) of reduced-fat dairy intakes (s/d) at age 10 y and excess adiposity at 13 y *^1^* | | | | | | |
| --- | --- | --- | --- | --- | --- | --- |
|  |  | Reduced-fat dairy intakes at 10 y, s/d | | | | *P* for trend |
| Excess adiposity at age 13 y |  | C1 | C2 | C3 | C4 |  |
|  |  | 0.04 ± 0.09  (0 - 0.22) *^2^* | 0.45 ± 0.15  (0.21 - 0.70) | 0.89 ± 0.20  (0.60 - 1.24) | 1.78 ± 0.62  (1.08 - 3.11) |  |
|  |  | *n*=884 | *n*=525 | *n*=524 | *n*=522 |  |
| Excess fat mass | *n* [%] | 181 [21] | 105 [20] | 102 [19] | 103 [20] |  |
|  |  |  | OR (95% CI) | OR (95% CI) | OR (95% CI) |  |
| Model 1 |  | 1.00 | 0.85 (0.58, 1.24) | 0.82 (0.55, 1.22) | 0.84 (0.56, 1.27) | 0.33 |
| Model 2 |  | 1.00 | 0.85 (0.58, 1.26) | 0.86 (0.58, 1.30) | 0.85 (0.55, 1.30) | 0.39 |
| Model 3 |  | 1.00 | 0.88 (0.59, 1.31) | 0.88 (0.58, 1.34) | 0.88 (0.56, 1.39) | 0.53 |
| Model 4 |  | 1.00 | 0.86 (0.58, 1.29) | 0.87 (0.57, 1.33) | 0.93 (0.59, 1.47) | 0.65 |
| Model 5 |  | 1.00 | 0.86 (0.58, 1.28) | 0.85 (0.55, 1.29) | 0.90 (0.56, 1.44) | 0.52 |
| Overweight | *n* [%] | 170 [19] | 107 [20] | 99 [19] | 107 [21] |  |
|  |  |  | OR (95% CI) | OR (95% CI) | OR (95% CI) |  |
| Model 1 |  | 1.00 | 0.99 (0.65, 1.51) | 0.83 (0.53, 1.28) | 0.98 (0.62, 1.55) | 0.75 |
| Model 2 |  | 1.00 | 1.01 (0.66, 1.54) | 0.84 (0.54, 1.32) | 1.01 (0.63, 1.60) | 0.84 |
| Model 3 |  | 1.00 | 1.01 (0.66, 1.55) | 0.81 (0.51, 1.30) | 1.01 (0.62, 1.64) | 0.81 |
| Model 4 |  | 1.00 | 1.00 (0.65, 1.54) | 0.82 (0.51, 1.31) | 1.03 (0.63, 1.69) | 0.88 |
| Model 5 |  | 1.00 | 0.98 (0.64, 1.52) | 0.78 (0.49, 1.25) | 0.91 (0.55, 1.52) | 0.54 |
| *^1^* Consistent with the USDA, a serving of dairy was defined as a calcium equivalent to 1 cup of milk ([21](#_ENREF_21)). The lowest category (C1) of reduced-fat dairy intake (s/d) was defined as the bottom 36% of consumers due to a large number of participants with no consumption of reduced-fat dairy products at age 10 y (as much as 36% in some sex- and BMI-specific strata). The remaining participants were categorized using tertiles (C2 to C4). Excess fat mass was defined as the top quintile for sex-specific and age- and height-adjusted total body fat mass (kg) and overweight using International Obesity Taskforce age- and sex-specific weight categories ([27](#_ENREF_27)). Multivariable logistic regression (proc logistic in SAS) was used to examine the effects of servings per day (s/d) of dairy intake on the odds of excess adiposity. *P* for trend was determined by treating levels of intake as a continuous variable. **Model 1** (simple) - age 10 y, sex, height 10 y, reduced-fat dairy 13 y (categorical), full-fat dairy 10 and 13 (categorical) and adiposity 10 y (continuous); **Model 2** (demographics) – model 1 plus maternal education and overweight status, physical activity13 y, pubertal stage13 y and dieting 13 y; **Model 3** (diet) – model 2 plus 10 y intakes of fruit juice, fruits and vegetables, total fat, total protein, sugar-sweetened beverages, fiber, and cereal; **Model 4** (reporting errors) – model 3 additionally adjusted for dietary reporting errors 13 y.  **Model 5** (energy adjusted) – model 4 plus adjustment for total dairy intakes.  *^2^* Values represent the mean ± SD and (5^th^ - 95^th^ percentiles) of reduced-fat dairy intakes at age 10 y in s/d. | | | | | | |
